# Study of the Direct Red 81 Dye/Copper(II)-Phenanthroline System

**DOI:** 10.3390/molecules23020242

**Published:** 2018-01-25

**Authors:** Elsa Walger, Nathalie Marlin, Florian Molton, Gérard Mortha

**Affiliations:** 1 Univ. Grenoble Alpes, CNRS, Grenoble INP, LGP2, 461 rue de la Papeterie—CS 10065, 38402 Saint Martin d’Hères Cedex, F-38000 Grenoble, France; nathalie.marlin@pagora.grenoble-inp.fr (N.M.); gerard.mortha@pagora.grenoble-inp.fr (G.M.); 2 Univ. Grenoble Alpes, CNRS, DCM, CS 40700, 38058 Grenoble Cedex 9, F-38000 Grenoble, France; florian.molton@univ-grenoble-alpes.fr

**Keywords:** Direct Red 81 azo dye, copper(II)-phenanthroline, UV-vis spectroscopy, coordination, EPR spectroscopy, speciation simulation, color-stripping

## Abstract

Recovered papers contain several chromophores, such as wood lignin and dyes. These have to be eliminated during paper recycling in order to produce white paper. Hydrogen peroxide under alkaline conditions is generally used to decolorize lignin, but its effect on dyes is limited. Copper(II)-phenanthroline (Cu-Phen) complexes can activate the oxidation of lignin by hydrogen peroxide. Hydrogen peroxide may also be activated during recycled fiber bleaching, thus enhancing its color-stripping efficiency towards unoxidizable azo dyes. The purpose of this paper was to determine the effect of Cu-Phen complexes on a model azo dye, Direct Red 81 (DR81), in aqueous solution. Different Cu-Phen solutions (with different initial Cu:Phen molar ratios) were prepared and mixed with the dye at different pHs. The geochemical computer program PHREEQC allowed precise calculation of the theoretical distribution between different possible coordinates (CuPhenOH^+^, Cu(Phen)_2_^2+^, CuPhen(OH)_2_, Cu(Phen)_3_^2+^, etc.) depending on pH and initial concentrations. UV-vis spectroscopic measurements were correlated with the major species theoretically present in each condition. The UV absorbance of the system was mainly attributed to the Cu-Phen complex and the visible absorbance was only due to the dye. Cu-Phen appeared to reduce the color intensity of the DR81 dye aqueous solution under specific conditions (more effective at pH 10.7 with Cu:Phen = 1:1), probably owing to the occurrence of a coordination phenomenon between DR81 and Cu-Phen. Hence, the ligand competition between phenanthroline and hydroxide ions would be disturbed by a third competitor, which is the dye molecule. Further investigation proved that the DR81 dye is able to form a complex with copper-phenanthroline, leading to partial color-stripping. This new “color-stripping effect” may be a new opportunity in paper and textile industries for wastewater treatment.

## 1. Introduction

Dyes are commonly used in paper manufacturing as additives to produce colored papers. They absorb the visible light because of their highly conjugated structures, which contain several groups with π-electron clouds, such as azo groups, ethylene groups, carbonyl groups and aromatic rings. When combined, these structures constitute the chromophore part of the molecule. The auxochrome groups provide the other properties of the dye: solubility, charge, and affinity to cellulose. These are amine, hydroxyl, sulfonic or carboxyl groups.

**Figure 1:**
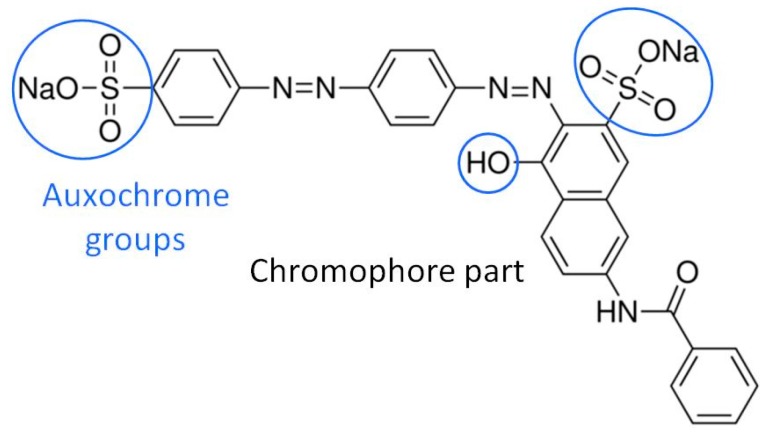
Molecular structure of Direct Red 81 dye.

Direct dyes have a good affinity with both bleached and unbleached chemical pulps. They represent more than half of the dyes present in the pulp and textile industries [1] and they usually contain at least one azo group and some sulfonic acid groups thus making them soluble. Most dyes used in the industry are azo dyes, such as the Direct Red 81 dye (Figure 1).

Consequently, recovered papers contain several chromophores: lignin from mechanical pulp or highly colored Kraft pulps, and also dyes. These chromophores have to be eliminated during paper recycling in order to produce white paper. In industrial recycling lines, the bleaching treatment, following the deinking operation, usually consists of several steps using different oxidant or reducing chemicals. In this context hydrogen peroxide is used under alkaline conditions to decolorize lignin. Its anionic form, the perhydroxyl anion HOO^−^ (pKa = 11.6 at 25 °C), oxidizes the conjugated carbonyl groups of lignin into carboxylic acid groups, leading to higher pulp brightness. However, its effect is limited on dyes because they mainly contain unsaturated carbon-carbon bonds and unreactive azo functions. Therefore, it is often combined with a reducing agent such as sodium dithionite or formamidinesulfinic acid (FAS), although these are more expensive, in order to reach better final brightness [2]. Indeed, unlike hydrogen peroxide, these reductive chemicals are quite efficient for reduction of azo bonds and consequently dye color-stripping.

Since azo bonds are not oxidizable by HOO^−^, direct azo dyes are not fully degraded by conventional hydrogen peroxide bleaching stages. Therefore, in order to reduce the use of sodium dithionite and FAS in recycling lines, the idea would be to improve the decolorizing action of hydrogen peroxide by activation or catalysis.

**Figure 2:**
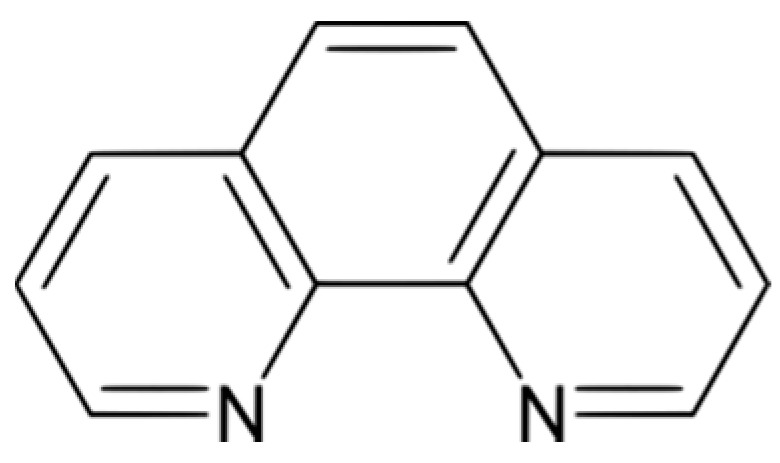
Molecular structure of 1,10-phenanthroline (Phen).

2,2′-Bipyridine was synthesized by Fritz Blau in 1888 and discovered to form intense red ferrous salts. In 1898, he synthesized the 1,10-phenanthroline molecule (Figure 2) and showed that its properties were similar to those of 2,2′-bipyridine. He prepared iron, nickel, cobalt and copper(II) complex salts of 2,2′-bipyridine and 1,10-phenanthroline and was able to recognize them as Werner coordination compounds, with an octahedral configuration and so classed them as hexaammines [3].

In 1979, Cu^+^(phenanthroline)_2_ was discovered to have a nuclease activity [4], which means that it is able to cleave the phosphodiester bonds between DNA or RNA nucleotides. The scission of DNA is supposed to occur in the presence of two co-reactants: Cu^+^(phenanthroline)_2_ and H_2_O_2_ [5,6]. According to Sigman [5], Cu^+^(phenanthroline)_2_ would bind to DNA (1) and the oxidation of this coordinate by H_2_O_2_ would form a cupric hydroxyl radical-like coordinate (2), responsible for the scission of DNA (3) into oligonucleotides.

$$DNA+Cu^{+}{\left(Phen\right)}_{2}\overset{\;\left(1\right)\;}{\to }DNA\cdots Cu^{+}{\left(Phen\right)}_{2}\overset{\begin{array}{c} \left(2\right)\\ H_{2}O_{2} \end{array}}{\to }DNA\cdots HO\;˙Cu^{2+}{\left(Phen\right)}_{2}$$

$$\overset{\left(3\right)\;}{\to }oligonucleotide\;products$$

Copper-phenanthroline coordinates (Cu-Phen) were thus the first artificial nucleases studied in biology. They could be either bis(1,10-phenanthroline)copper as described by Sigman [4,5,6] or mono(1,10-phenanthroline)copper [7].

This oxidative action of Cu-Phen complexes in the presence of H_2_O_2_ has been the starting point of oxidation studies of lignin compounds using copper-phenanthroline coordinates. Previous works conducted with such coordinates have reported the activation of H_2_O_2_ for chemical pulp delignification in alkaline conditions [8,9,10], leading to patents [11,12]. The results depended on the complex formulation, with an initial copper-to-phenanthroline molar ratio Cu:Phen of 1:1, 1:2 or 1:3. Most papers have focused on the 1:2 ratio. They have shown that the action of H_2_O_2_ could be enhanced thanks to Cu-Phen complexes. Several works have also been conducted on the catalytic action of Cu-Phen for the alkaline oxidation of lignin by either oxygen [13,14,15,16,17] or hydrogen peroxide [9,17], especially with lignin model compounds such as veratryl alcohol. In this context, the distribution of different copper-phenanthroline-hydroxyl complexes, depending on the pH and initial concentrations of copper sulfate and phenanthroline, was described by Korpi [14]. This distribution was calculated thanks to the HySS program [18], using stability constants from the NIST Standard Reference Database 46.

With a view to applying this activated or catalyzed hydrogen peroxide chemical stage to recycled fiber bleaching, some trials with deinked pulps and colored pulp were also conducted under alkaline conditions [9,19,20]. These showed that the dye removal index, indicating the loss of color in the CIE L*a*b* color space as defined by Sharpe and Lowe [21], was improved during a peroxide stage activated with a copper-phenanthroline complex.

This finding indicates that Cu-Phen could make hydrogen peroxide effective in dye color-stripping although it does not decolorize dyed pulps under classical conditions. 

This activation phenomenon is complex since it involves a liquid-solid system and several possible effects: (1) oxidation of the dye by H_2_O_2_ alone; (2) chemical action of Cu-Phen itself on the dye molecule; (3) activation of H_2_O_2_ by Cu-Phen to oxidize the dye; and (4) oxidation of the dye by free radicals originated from H_2_O_2_ decomposition in the presence of Cu-Phen. All these effects were studied in detail in the work of Walger [22].

In the present paper, the focus was only on the dye/copper-phenanthroline system without any oxidant, particularly as the results were already interesting in terms of color-stripping. The Direct Red 81 dye, a commercial dye solution used in the paper industry, was selected as a model compound for hardly alkaline H_2_O_2_-oxidizable paper dyes present in recovered fibers. The chemical action of copper-phenanthroline complexes on Direct Red 81 in aqueous solution, in the absence of fibers, was thus examined. Since most of the previous studies only focused on alkaline pH with the aim of catalyzing alkaline peroxide stages, the effect of pH was also investigated in the present study. Copper-phenanthroline speciation using the PHREEQC software was followed as a function of pH and Cu:Phen ratio. The Cu-Phen/DR81 system was investigated using UV-vis spectroscopy, Cu-Phen speciation and Electron Paramagnetic Resonance (EPR) spectroscopy for the detection of copper(II) and its molecular environment.This study should contribute to the development of new wastewater treatments in the paper and textile industries as well as that of deinked pulp bleaching.

## 2. Results and Discussion

UV-visible spectroscopy is a convenient method to characterize dyes. Dye structures and their possible modifications, due to variations of physicochemical properties of the medium or due to reactions, can be detected. The following discussion is thus supported by UV-vis analyses of the dye solution with or without Cu-Phen. Controls without Direct Red 81 were also studied for comparison.

### 2.1. UV-Visible Analysis of the Dye

As shown in Figure 1, the DR81 molecule bears two azo groups and five aromatic nuclei, which are conjugated and constitute the chromophore part, while the auxochrome groups consist of one hydroxyl group (of the naphthol group) and two sulfonic acid groups that allow the dissolution of DR81 into water and its binding to cellulosic fibers. The dye molecule, thermally stable (no degradation at 70 °C), was first analyzed alone at different pHs.

**Figure 3:**
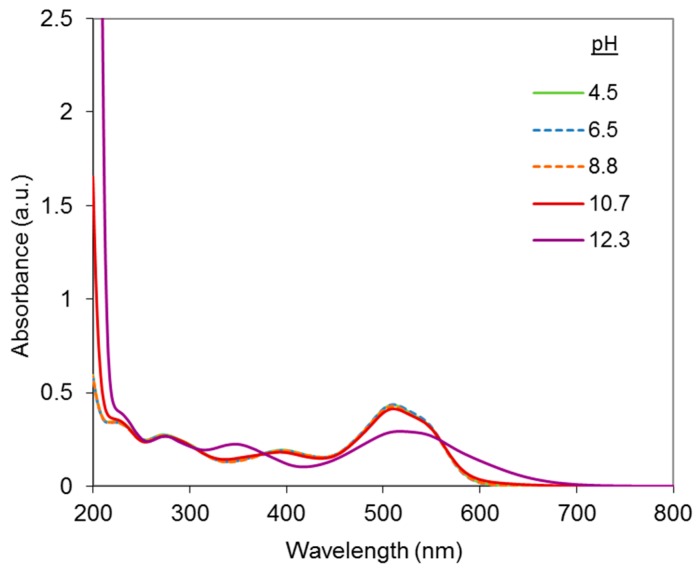
UV-vis spectra of the DR81 dye diluted in water (10 µM), at different pHs.

Figure 3 shows the UV-vis spectra of the DR81 dye at pH varying from 4.5 to 12.3, obtained with the addition of 1 M NaOH or diluted H_2_SO_4_.

In the visible range, the dye solution exhibited a maximal light absorbance at around 510–518 nm depending on the pH. It was red from pH 4.5 to pH 10.7 (λ_max_ around 510 nm) and red-purple at strong alkaline pH (12.3) (λ_max_ = 517.5 nm). This is illustrated in Figure 3 by the bathochromic and hypochromic absorbance shifts observed between pH 10.7 and pH 12.3.

The bathochromic shift can be attributed to the ionization of the free naphthol group of the dye. Since the acid dissociation constants of naphthol groups of common dyes are typically around or above 10, lower pHs were tested in order to determine more precisely the ionization pHs. According to Figure 3, there is almost no change of the spectrum between pH 4.5 and 10.7, which confirms that ionization takes place only between pH 10.7 and 12.3.

Therefore, in the further study on dye interactions with Cu-Phen, it was decided to focus on these two pH values (10.7 and 12.3) in order to study the ionized and non-ionized forms of DR81.

Before examining the dye-Cu-Phen system, the Cu-Phen complex was first studied using (1) speciation calculations to predict the concentration-pH profile of Cu-Phen species present in solution and (2) UV-vis spectroscopy to confirm the presence of coordinates and to examine how they are formed.

### 2.2. Copper-Phenanthroline Speciation

The composition of a metal ion-ligand solution at thermodynamic equilibrium depends on the initial reagent concentrations, temperature and pH [23]. With the copper(II)-1,10-phenanthroline system, various species can be formed, depending on the Cu:Phen stoichiometric ratio (1:1, 1:2, 1:3), typically free and hydroxylated copper, free phenanthroline (Phen) and hydroxylated or non-hydroxylated complexes such as CuPhen(OH)_2_, Cu(Phen)_3_^2+^, CuPhenOH^+^, Cu(Phen)_2_^2+^, etc. [24,25,26,27].

**Figure 4:**
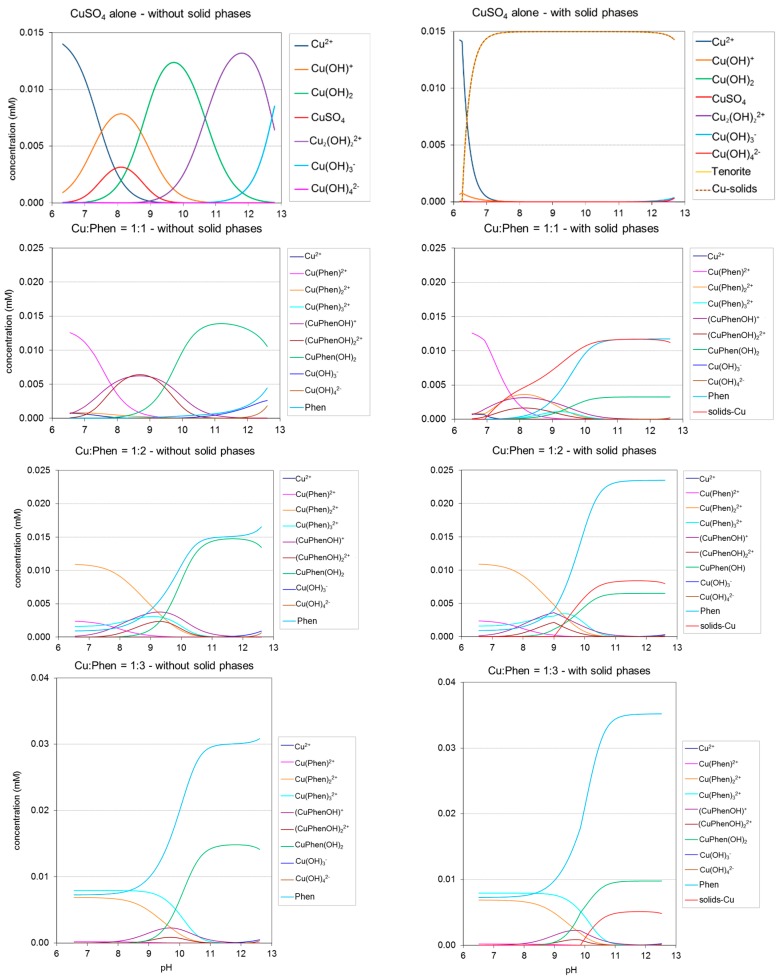
Calculated distributions of different species in CuSO_4_ and Cu-Phen solutions as a function of pH. CuSO_4_ alone, Cu:Phen = 1:1, 1:2 and 1:3; initial CuSO_4_ concentration [Cu] = 15 µM at 25 °C, with or without the occurrence of precipitation.

Modeling using the PHREEQC software allowed the calculation of equilibrium concentration-pH profiles for each Cu-Phen species including free copper and free phenanthroline, with or without considering the occurrence of coordinate precipitation. The predicted results are shown in Figure 4. They were calculated for Cu:Phen = 1:1, 1:2 and 1:3 with an initial CuSO_4_ concentration of 15 µM at 25 °C, from pH 6.5 to pH 12.5. In the case “with solid phase”, precipitation is made possible when the solubility product for one complex is attained. In the alternative case, entitled “without solid phases”, all species remain soluble and a certain degree of over-saturation is thus admitted for some of them (which allows considering cases where precipitation occurs very slowly at a certain degree of over-saturation). In the “with solid phase” case, total solids are represented by one profile gathering every copper-based precipitate theoretically present.

Without solid phase, under the calculation conditions, copper sulfate gave rise to a majority of free cupric ions from pH 6 to 7.5. Increasing the pH raised the degree of hydroxylation of copper, reaching a majority of Cu(OH)_3_^−^ above pH 12.7. With the occurrence of precipitation, cupric ions appeared to be replaced by tenorite (CuO) from pH 6 to 7.4. Above 7.4, tenorite should be the only copper species present until pH 12.

In the case of Cu:Phen = 1:1, the calculated distributions were much different with or without solid phase. In the case “with solid phase”, most of the copper precipitates, starting at pH 7 and reaching a maximum (0.012 mM) above pH 10, leading to a high amount of free phenanthroline in solution. The experimental observation of the prepared mixtures confirmed this statement, since the Cu-Phen (1:1) stock solution was actually not stable and precipitates were visible after some days or weeks at rest.

Concerning the other Cu:Phen molar ratios (1:2 and 1:3), the modeling “with solid phase” resulted in lower amounts of precipitates (around 0.008 mM above pH 10.5 and 0.005 mM above pH 11, respectively, compared to 0.012 mM at a molar ratio of 1:1). Indeed, experimentally, these solutions were found more stable since no precipitate was visible after several weeks. Therefore, it appears that higher Phen:Cu molar ratios favor the existence of soluble complexes between copper and phenanthroline, rather than precipitated complexes, and that the calculated results are in general accordance with visual observations.

The distributions presented in Figure 4 differ from those described in the literature by Korpi et al. [14] since the initial concentrations (chosen to fit our experimental range) were much lower: [Cu] = 15 µM vs. 10 mM. The conditions of Korpi et al. were also tested for comparison and a good fit was found when the calculations did not allow precipitation. This shows the advantage of using the PHREEQC software, which takes into account the occurrence of solid precipitates and their physiochemical equilibria with dissolved species in the medium.

For further use in the investigation of Cu-Phen interaction with the dye at different pHs, three pH values were focused on: 6.5 (unionized dye at near-neutral pH), 10.7 (unionized dye at alkaline pH), and 12.3 (ionized dye at strongly alkaline pH). The abundances of the species at these specific pHs are gathered in Table 1 (without precipitation) and Table 2 (with precipitation) as a function of the initial Cu:Phen molar ratio.

According to Figure 4 and Table 1 and Table 2, two main features arise: (1) CuPhen(OH)_2_ appears to be the major copper species at alkaline pH (81 to 98% of total Cu in the case “without precipitation”) and (2), free phenanthroline is still present in the medium. As expected, free phenanthroline concentration (last row in Table 1) increases significantly when the Cu:Phen ratio varies from 1:1 to 1:3. Besides, more hydroxylated species are logically found at pH 12.3 vs. 10.7. Whatever the pH, Cu(Phen)^2+^ is almost inexistent, and the other non-hydroxylated species Cu(Phen)_2_^2+^ and Cu(Phen)_3_^2+^ are only present at pH 10.7, at low concentrations.

At near neutral pH (6.5), copper and phenanthroline mainly form Cu(Phen)^2+^, Cu(Phen)_2_^2+^ and Cu(Phen)_3_^2+^, at relative percentages depending on the Cu:Phen molar ratio.As explained before, when the Cu-Phen stock solution with a molar ratio of 1:1 was stored for several days, the solution eventually precipitated. Therefore, all the experiments that will be described in the next paragraphs were performed with freshly prepared solutions whatever the Cu:Phen molar ratio, and no precipitation was observed at initial time. Consequently, all explanations will be referred to predictions “without solids”.

### 2.3. Analysis of the Cu-Phen Complex by UV-Vis Spectroscopy

The Cu-Phen complexation was mainly analyzed by UV-visible spectroscopy. The study was conducted at alkaline pH (12.3), i.e., the pH at which the complex is supposed to be used for H_2_O_2_ color-stripping activation. Some analyses were also carried out at pH 10.7 (pH of dye ionization) for comparison. The contribution of CuSO_4_ and Phen alone to the UV-vis absorbance was first analyzed.

#### 2.3.1. CuSO_4_

**Figure 5:**
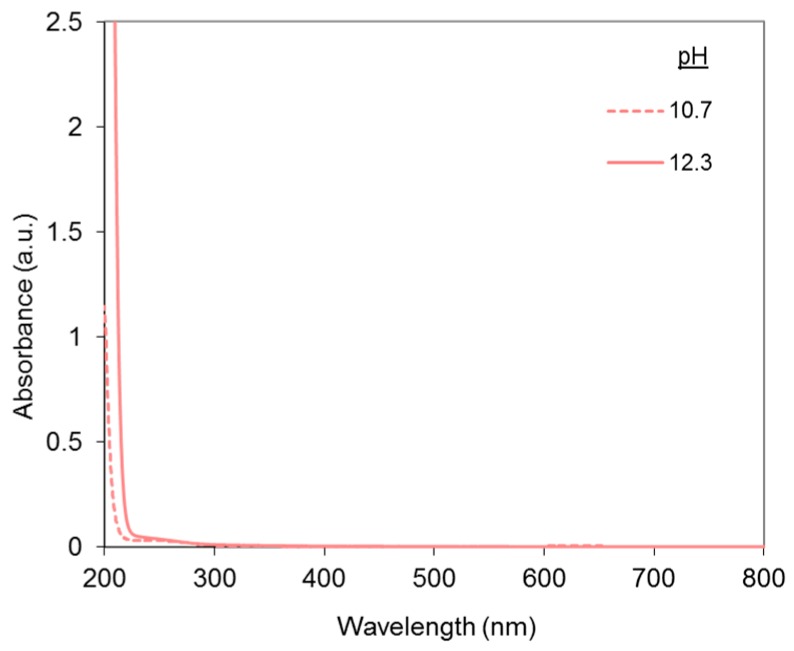
Absorbance spectra of copper sulfate solutions at pH 10.7 and 12.3, with [CuSO_4_] = 15 µM.

Copper sulfate absorbance spectra at pH 10.7 and 12.3 are shown in Figure 5. 

At the low concentration used in this study, a slight absorption contribution can be noticed in the UV range and no absorbance is detected in the visible range. Yet, the blue shade of copper(II) is visible at higher concentrations.

#### 2.3.2. Phenanthroline

**Figure 6:**
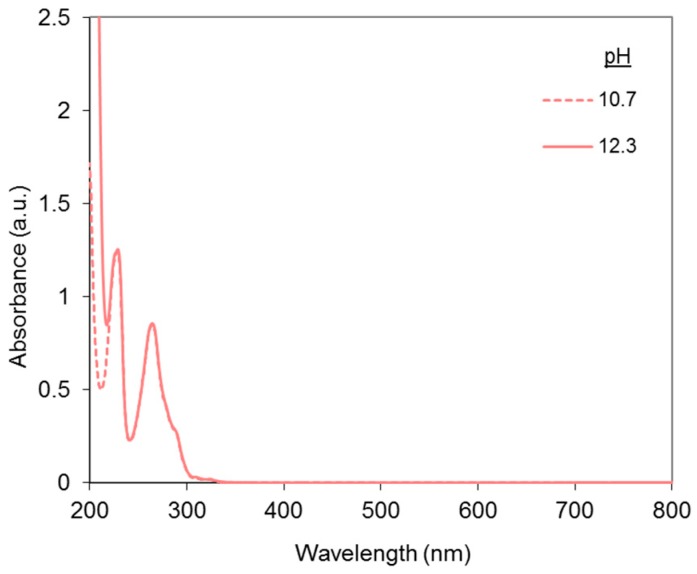
Absorbance spectra of 1,10-phenanthroline at pH 10.7 and 12.3, with [Phen] = 30 µM (equivalent to the amount of phenanthroline with the following molar ratio: DR81:Cu = 1:1.5, Cu:Phen = 1:2, and [Cu] = 15 µM).

The UV-vis absorbance spectra of phenanthroline alone at pH 10.7 and 12.3 are presented in Figure 6.

Two peaks are observed at 228.5–229 nm and 264 nm and nothing appears in the visible range. This is in accordance with previous results from Yamazaki et al. [28], who reported the first peak around 227 nm and the second peak at 263 nm, at near-neutral pH in solutions containing hexane. Therefore, phenanthroline’s absorbance will not interfere with the dye’s signal in the visible range. The same authors reported molar extinction coefficients at various wavelengths: log ε_max_ (227 nm) = 5.3; log ε_max_ (263 nm) = 5.2. In the present work, slightly different values were found: log ε_max_ (228.5 nm) = 4.6, and log ε_max_ (264 nm) = 4.5, probably attributable to differences of analytical conditions (different solvents and pHs). However, the result for the second peak is very close to that of Armaroli et al. [29] in a CH_2_Cl_2_ solution (log ε_max_ (264 nm) = 4.5), and to that of Vallée et al. [30] with crystalline dihydrochloride phenanthroline (log ε_max_ (265 nm) = 4.5). It can also be noticed that the phenanthroline spectra presented in Figure 6 were identical at both pHs except from 200 to 220 nm, where the absorbance of sodium hydroxide interfered.

#### 2.3.3. Cu-Phen

**Figure 7:**
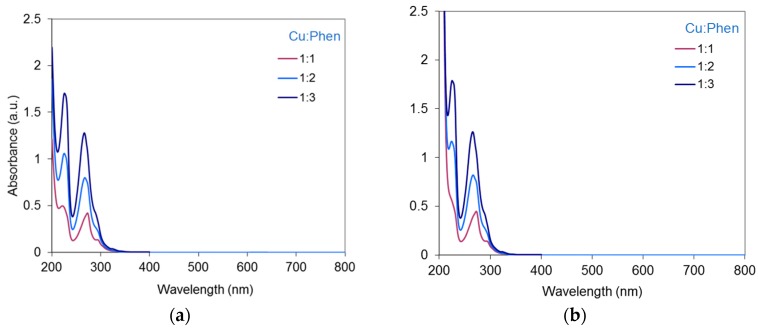
Absorbance spectra of the Cu-Phen solutions at (**a**) pH 10.7 and (**b**) pH 12.3, with Cu:Phen molar ratio = 1:1, 1:2 and 1:3, and [Cu] = 15 µM.

The absorbance spectra of Cu-Phen solutions at pH 10.7 and pH 12.3 are presented in Figure 7 with Cu:Phen molar ratios varying from 1:1 to 1:3.The Cu-Phen spectra are very close to those of Phen alone (Figure 6). This was expected since Ni et al. reported two peaks at 227 nm and 264.5 nm for Cu(Phen)_2_^2+^ prepared with Cu(NO_3_)_2_ and 1,10-phenanthroline monohydrate in a tris-HCl buffer (pH 7.4) [31]. Figure 7 indicates that at each pH, the absorbance of copper-phenanthroline increases almost proportionally to the initial concentration of phenanthroline in the complex, in accordance with Beer-Lambert’s law. As observed with Phen alone, at the concentrations used in the present study, Cu-Phen solutions did not exhibit any signal in the visible range. However, at various pHs and Cu:Phen molar ratios, slight differences between Phen and Cu-Phen can be observed in terms of maximum absorption wavelengths, showing the effect of coordination between copper and phenanthroline. Moreover, no clear difference was observed between pH 10.7 and 12.3, suggesting that coordination was not influenced by pH, probably because at these pHs, the major copper species is CuPhen(OH)_2_. In depth examination of Cu-Phen coordination by EPR spectroscopy will be presented in the following section.

#### 2.3.4. Evidence of Cu-Phen Coordination by EPR

**Figure 8:**
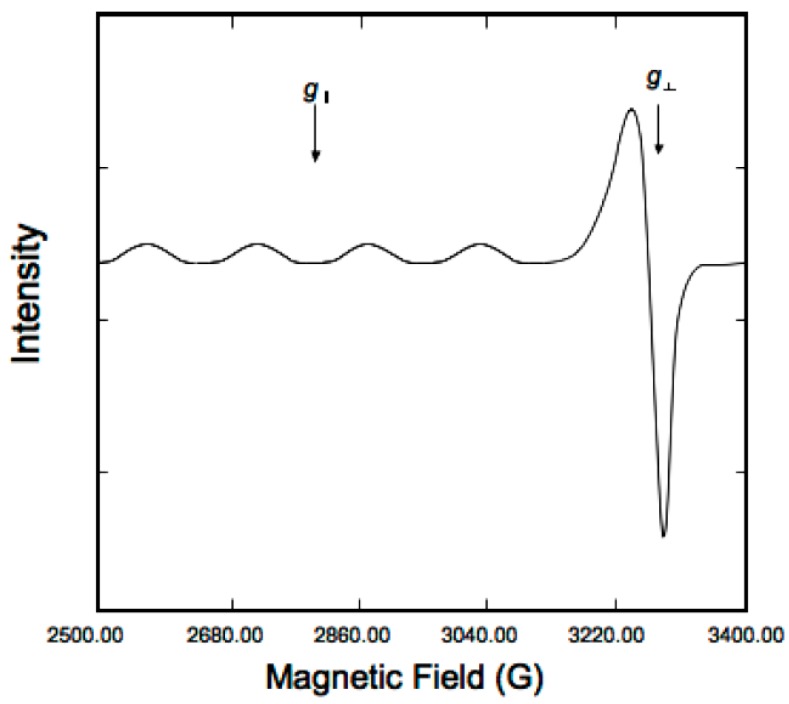
Typical X-band EPR spectrum of copper(II) compounds [32].

Paramagnetic species such as copper(II) are detectable by EPR spectroscopy, especially at very low temperatures, at which hyperfine structures can be observed. An example of this is shown in Figure 8. The technique and experimental conditions are presented in Section 4.5.

**Figure 9:**
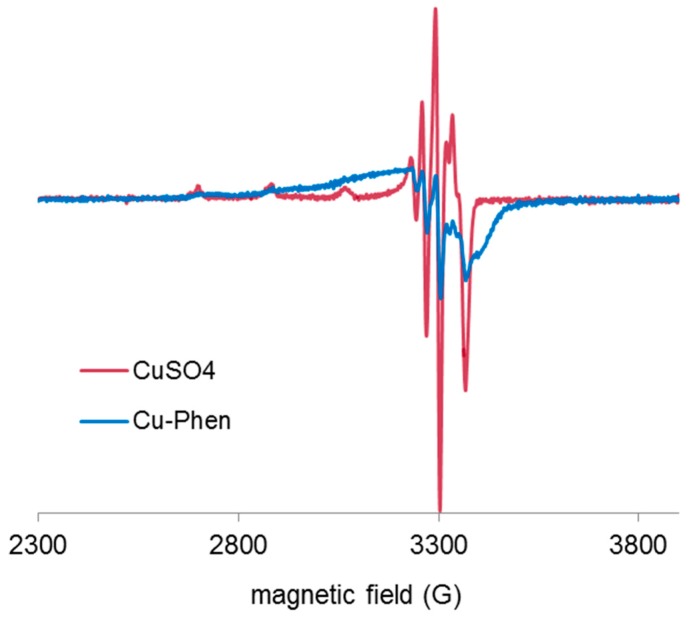
Experimental X-band EPR spectra recorded at 100 K in frozen aqueous solutions of CuSO_4_ and Cu-Phen at pH 12.3, with Cu:Phen molar ratio = 1:2 and [Cu] = 1.5 mM.

X-band EPR spectra of the Cu salt (CuSO_4_) and of Cu-Phen were recorded at 100 K and at pH 12.3 (Figure 9). EPR detection of copper(II) requiring millimolar concentration, these analyses were performed with [Cu] = 1.5 mM (100 times higher than for the UV experiments).

Both EPR spectra are characteristic of *S* = ½ systems and consistent with the presence of mononuclear paramagnetic Cu(II) (a d^9^ ion), since Cu(I) (a d^10^ ion) is diamagnetic. It is therefore evidenced that Cu is present in solution in its oxidized state, Cu^2+^, and that the presence of phenanthroline does not affect its degree of oxidation. However, when phenanthroline is present in solution, the EPR spectrum is significantly modified, which shows a strong modification of the copper environment due to the coordination between Cu^2+^ and phenanthroline. The effect of adding the dye is presented in the next paragraph.

### 2.4. Analysis of the DR81 Dye/Cu-Phen System

The possibility of dye complexation with CuSO_4_ and Cu-Phen was first examined by UV-vis spectroscopy. The study was mainly conducted at strong alkaline pH since the Cu-Phen complex is supposed to be used as an activator of alkaline H_2_O_2_ color-stripping treatments for dyed cellulosic fibers. Some investigations were also performed at lower pH for a better understanding of the phenomena. Before studying the complete three-component system, several controls were performed with dye-copper alone and dye-phenanthroline alone.

#### 2.4.1. Dye/CuSO_4_ System

**Figure 10:**
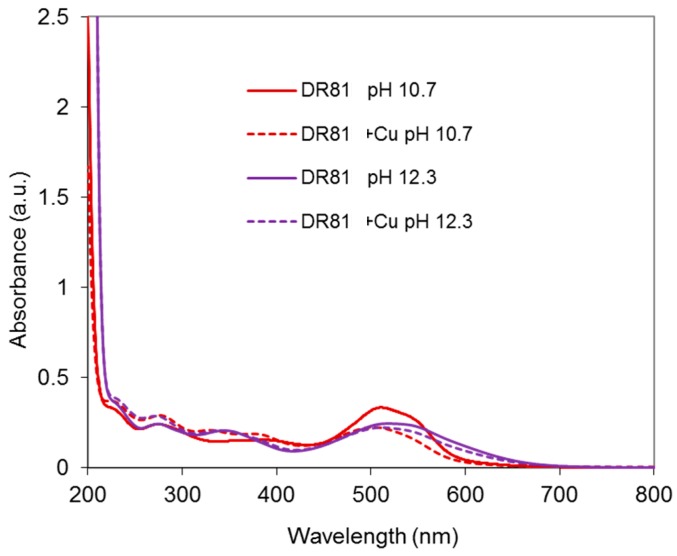
Absorbance spectra of the dye/copper sulfate system at pH 10.7 and 12.3, with DR81:CuSO_4_ molar ratio= 1:1.5 and [DR81] = 10 µM, compared to the dye alone at the same pHs.

As a first control, CuSO_4_ was introduced at alkaline pH in the dye solution at a dye:Cu molar ratio of 1:1.5. Results are presented in Figure 10. The dye is responsible for the total absorbance in the visible range. Adding CuSO_4_ to the dye solution modifies the absorbance, especially at pH 10.7, with a strong hypochromic effect (33% absorbance decrease at 510 nm), which evidences a significant modification of the dye chromophore structure due to coordination.

#### 2.4.2. Dye/Phenanthroline System

**Figure 11:**
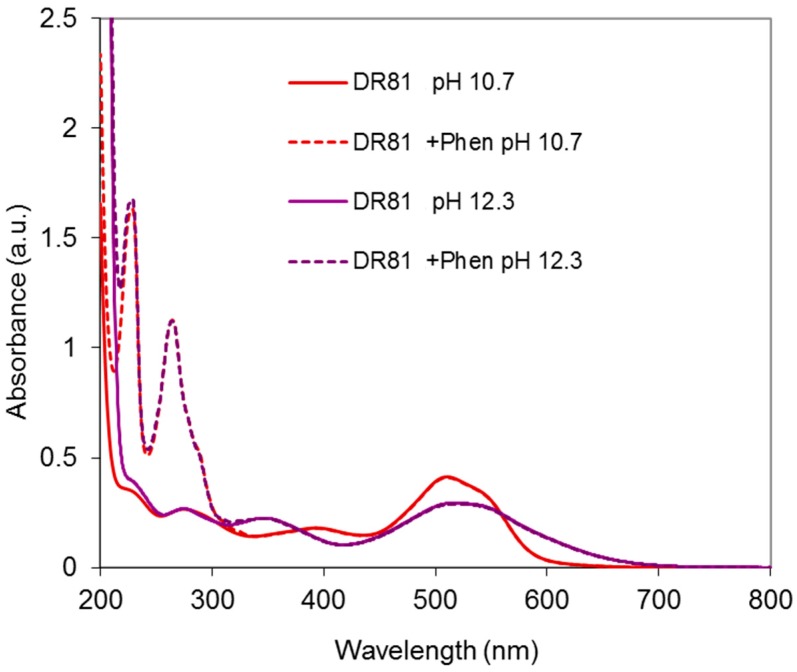
Absorbance spectra of the dye/phenanthroline system at pH 10.7 and 12.3, with DR81:Phen molar ratio = 1:3 and [DR81] = 10 µM (equivalent to the amount of phenanthroline with DR81:Cu molar ratio = 1:1.5, Cu:Phen molar ratio = 1:2, and [Cu] = 15 µM), compared to the dye alone at the same pHs.

As a second control, phenanthroline alone was added to the dye solution. The resulting spectra are presented in Figure 11. It is shown that the chromophore structure of the dye remains unchanged with or without phenanthroline. Hence, no interaction between phenanthroline and the dye is evidenced.

#### 2.4.3. Dye/Cu-Phen System

##### Results at Alkaline pH

**Figure 12:**
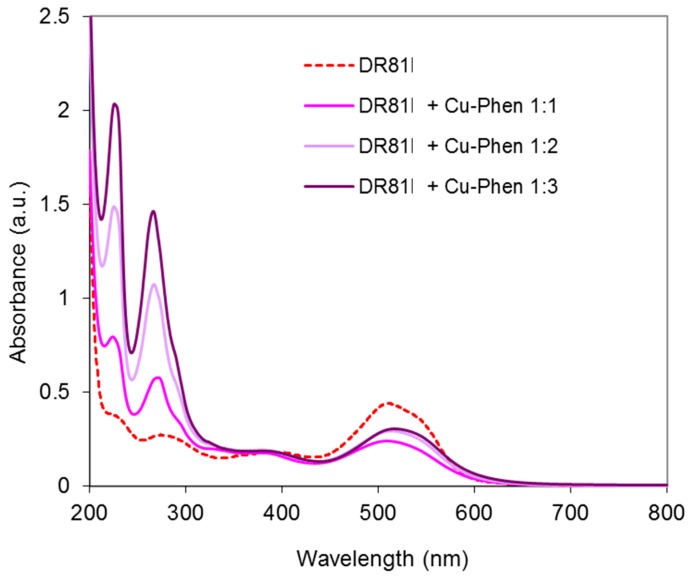
Absorbance spectra of the dye/copper-phenanthroline system at pH 10.7, with Cu:Phen molar ratio = 1:1, 1:2 and 1:3, DR81:Cu molar ratio= 1:1.5, and [DR81] = 10 µM, compared to the dye alone at the same pH.

The system was studied at pH 10.7 and 12.3 and at three Cu:Phen ratios, to see the effect of dye ionization and copper-phenanthroline species abundance, as presented earlier. Figure 12 displays the absorbance spectra obtained at pH 10.7. At this pH, the dye is not ionized. The spectrum of the dye/Cu-Phen solution appears quite different from that of the dye alone, especially in the visible range, with a strong hypochromic effect similar to that observed with CuSO_4_ alone (Figure 10). The absorbance decay reached 33% for Cu:Phen molar ratios of 1:2 and 1:3 and for pure CuSO_4_, whereas it reached 45% at 510 nm for Cu:Phen at 1:1 molar ratio, thus showing that almost 50% of “color stripping” effect can be obtained by simple addition of Cu-Phen in the dye solution.

In addition to the hypochromic effect, a bathochromic shift is observed in some cases: the maximum absorption wavelength of the dye at 510 nm was shifted to 513.5 nm in the presence of Cu:Phen at a molar ratio of 1:2, and to 517 nm at a molar ratio of 1:3. This evidences the coordination between dye and copper, even in the presence of phenanthroline. The varying bathochromic shifts for various Cu:Phen ratios show that copper-phenanthroline and copper-dye coordination are in competition. Copper binds most likely to nitrogen atoms of the dye, as it is observed on common blue dyes such as Direct Blue 86 or Reactive Blue 163. Consequently, the π-conjugation structure of the dye is modified, leading to a hypochromic effect in the visible range, resulting in partial color-stripping. It is also interesting to notice that the presence of a limited amount of phenanthroline (as in the 1:1 molar ratio) leads to better decolorizing than without or with an excess of phenanthroline. An explanation can be that a minimum of phenanthroline is necessary to solubilize copper and carry it in the dye environment, but an excess of phenanthroline in the copper sphere of coordination competes with dye coordination.

In conclusion, at pH 10.7, one or several Cu-Phen species would coordinate to the dye molecule via the Cu atom, since the latter can bind to the nitrogen lone pairs of an azo group. For instance, if Cu(Phen)(OH)_2_ (the major species present, as calculated by PHREEQC) coordinates to the dye molecule, it would form a DR81-Cu-Phen-OH co-coordinate with total or partial release of the hydroxyl groups and/or phenanthroline ligand, depending on the Cu:Phen molar ratio.

**Figure 13:**
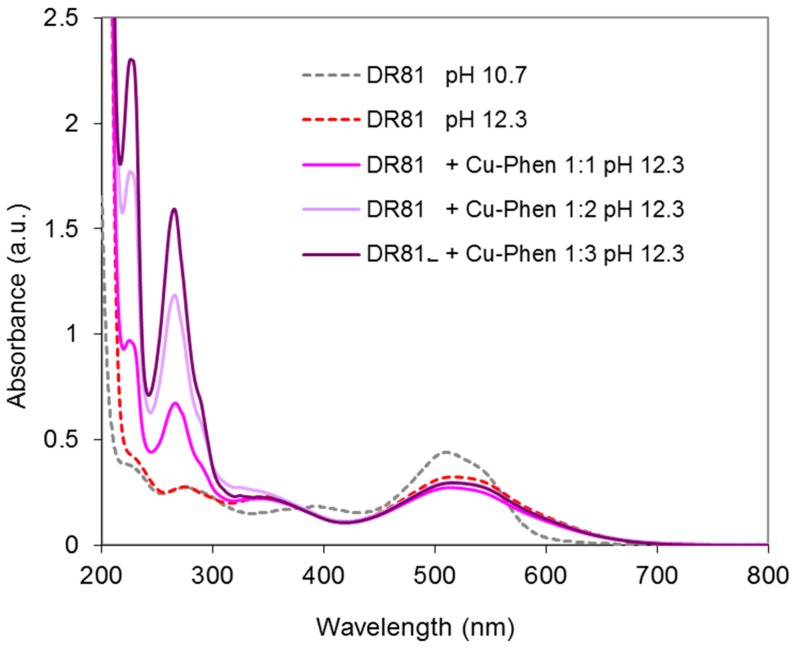
Absorbance spectra of the dye/copper-phenanthroline system at pH 12.3, with Cu:Phen molar ratio= 1:1, 1:2 and 1:3, DR81:Cu molar ratio= 1:1.5, and [DR81] = 10 µM, compared to the dye alone at pH 10.7 and 12.3.

The same trials were conducted at pH 12.3 (dye in ionized state). The results are given in Figure 13.

At pH 12.3, a hypochromic effect of lower magnitude was observed when adding Cu-Phen in the dye solution, by comparison to pH 10.7 (Figure 12). Again, this effect can be attributed to the complexation of copper with the dye in its ionized form. The weakest effect (9% absorbance decay) was observed for 1:2 and 1:3 Cu:Phen molar ratios (superimposed curves), of the same magnitude as with copper alone (CuSO_4_). Again at a Cu:Phen ratio of 1:1, complexation was more effective (17% absorbance decay). The same reasons as for pH 10.7 can be invoked.

The fact that dye color-stripping by Cu-Phen was more efficient when the dye was unionized may also be explained by the speciation calculations presented in Table 1. At pH 12.3, more hydroxylated copper species (Cu(OH)_3_^−^, Cu(OH)_4_^2−^, non-coordinated to phenanthroline) and more free phenanthroline are found, compared to pH 10.7, especially at a Cu:Phen ratio of 1:1. For the other Cu:Phen ratios, the amount of Cu(Phen)(OH)_2_ increases more significantly.

Overall, the results reveal better “color-stripping” effect at pH 10.7 than at pH 12.3, when the dye is unionized and copper less hydroxylated, inducing less competition for dye complexation in the copper coordination sphere. More, to “decolorize” the dye solution, CuSO_4_ was found as effective as Cu-Phen at molar ratios of 1:2 and 1:3, but less effective than Cu-Phen at a molar ratio of 1:1. Thus, a minimum amount of phenanthroline appears necessary to ensure copper dissolution, but at the same time, it should not limit dye complexation too much.

**Figure 14:**
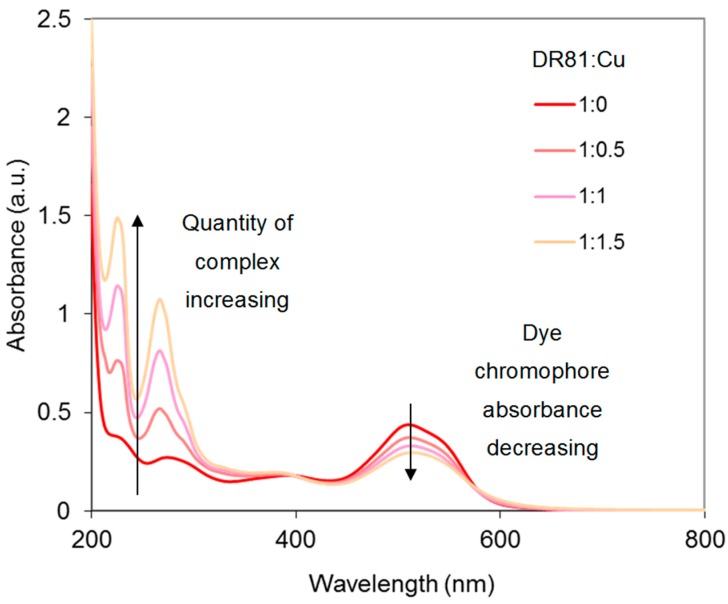
Absorbance spectra of the dye/copper-phenanthroline system at pH 10.7, with Cu:Phen molar ratio = 1:2 and [DR81] = 10 µM, for different DR81:Cu molar ratios.

**Figure 15:**
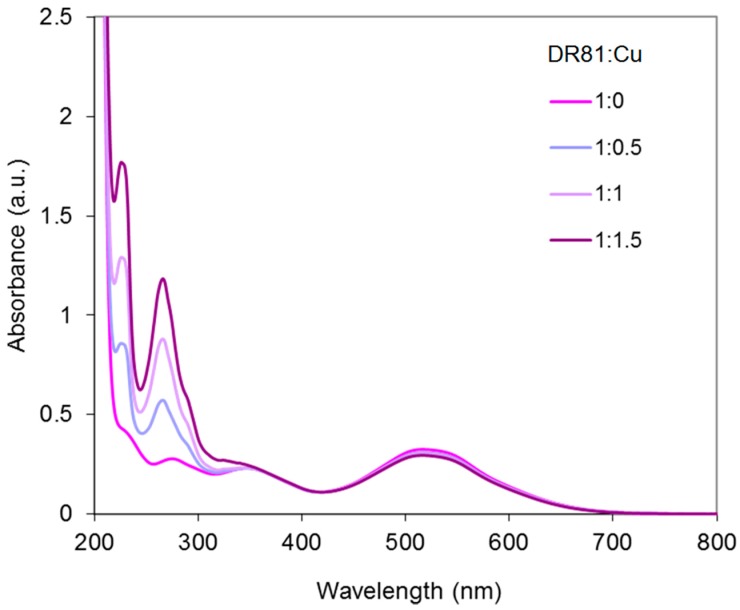
Absorbance spectra of the dye/copper-phenanthroline system at pH 12.3, with Cu:Phen molar ratio = 1:2 and [DR81] = 10 µM, for different DR81:Cu molar ratios.

Yet, for reproducible use, Cu-Phen solutions at a molar ratio of 1:1 were found too unstable and molar ratios of 1:2 and 1:3 were used in the next experiments, where the effect of the dye:Cu-Phen ratio was investigated. Results for tested ratios varying between 2:1 and 2:3, at pH 10.7 and 12.3, are presented in Figure 14 and Figure 15. At pH 10.7 (unionized dye), increasing the amount of Cu-Phen reduced significantly the dye absorbance, as a result of increased Cu-dye complexation. Total discoloration was not reached in these conditions and the absorbance decay remained lower than 50%. At pH 12.3 (ionized dye), the absorbance decrease was far less pronounced (almost inexistent). A Cu:Phen ratio of 1:3, not presented here, led to similar results.

As a summary, at pH 12.3, copper complexation has almost no effect and the change of dye color from neutral to alkaline solutions may only be attributed to ionization of the naphthol group of the dye, leading to bathochromic-hypochromic effects. An opposite case is observed at pH 10.7, where coordination between copper and dye are responsible for discoloration. Therefore, the presence of hydroxide ions and phenanthroline in the coordination sphere of copper plays a central role in the observed color-stripping effect, also related to the ionization of the dye at alkaline pH.

##### Results in Neutral Conditions

Since the dye complexation with Cu-Phen resulted in a stronger reduction of color in the case of pH 10.7 compared to pH 12.3, some tests were performed under near-neutral conditions, at pH 6.5. Controls were also carried out, excluding one component, before studying the complete three-component system (dye-Cu-Phen).

The first control test was a mixture of dye and CuSO_4_, in which case no precipitation occurred. Comparison with DR81 alone (results not presented) showed a very limited interaction between copper and dye. The second control was performed with pure phenanthroline. No interaction between the dye and phenanthroline occurred, as already seen at higher pHs.

**Figure 16:**
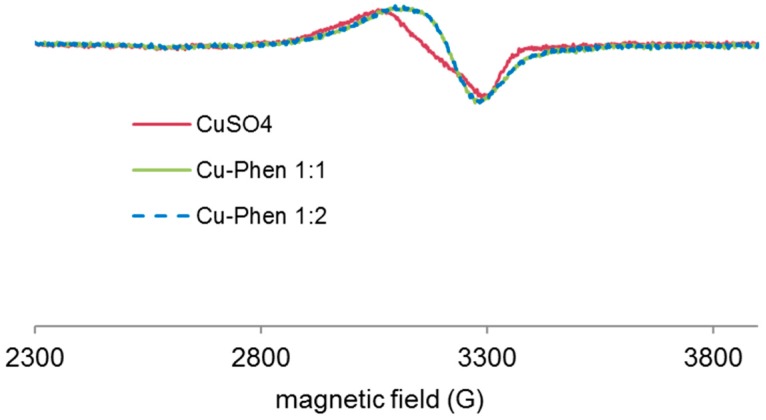
Experimental X-band EPR spectra recorded at 100 K in frozen aqueous solutions of CuSO_4_ and Cu-Phen at pH 6.5, with Cu:Phen molar ration = 1:1 and 1:2 and [Cu] = 1.5 mM.

Before studying the UV-vis response of the three-component system, coordination between copper and phenanthroline at near-neutral pH was verified. The EPR spectra obtained from frozen solutions of CuSO_4_ and Cu-Phen at pH 6.5 are presented in Figure 16.

At pH 6.5, the Cu-Phen signal (at 1:1 or 1:2 Cu:Phen molar ratio) was clearly different from that of CuSO_4_ alone, evidencing copper-phenanthroline coordination. Besides, since both Cu-Phen solutions exhibit the same signal (contrarily to the Cu-Phen signals observed at pH 12.3 in Figure 9), the major coordinates are the same in both cases. According to the speciation calculations by PHREEQC, with [Cu] = 1.5 mM, Cu(Phen)^2+^ would be the major species when Cu:Phen = 1:1 and Cu(Phen)_2_^2+^ would replace most of it when Cu:Phen = 1:2. Consequently, it can be hypothesized that the EPR signature of copper is not much modified whether one or two phenanthroline ligands are attached to the copper atom.

Besides, it can be verified that the CuSO_4_ signal in Figure 16 was different from that observed at alkaline pH in Figure 9, as it did not exhibit the multiple peaks around 3300 G attributed to copper hydroxylation. As a summary, it is experimentally verified here that Cu-Phen complexes are different at neutral and alkaline pH, as shown by the speciation calculations.

**Figure 17:**
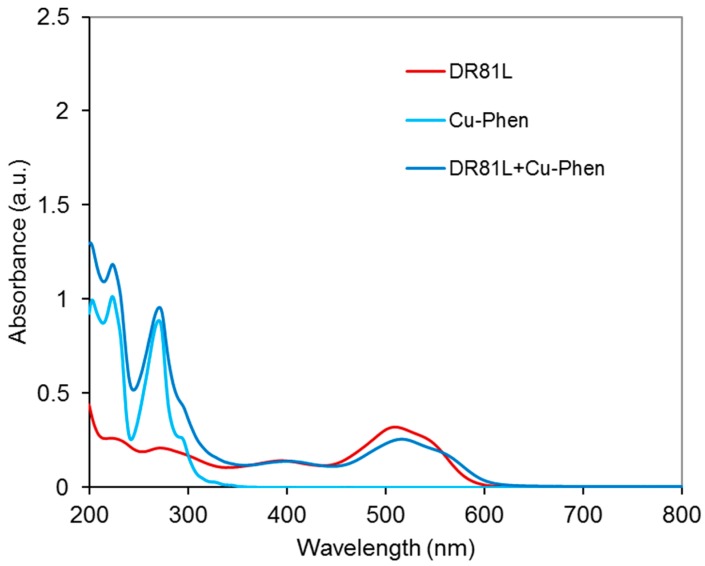
Absorbance spectrum of the dye/copper-phenanthroline system at pH 6.5, with Cu:Phen molar ratio = 1:2 and DR81:Cu molar ratio = 1:1.5, compared to the dye alone and copper-phenanthroline alone.

Figure 17 shows the UV-vis spectrum of the three-component system (Cu-Phen at 1:2 molar ratio) at pH 6.5. The absorbance of the dye is modified by the presence of Cu-Phen. The pronounced red shift (from 510 to 516.5 nm) and hypochromic effect (20% intensity decrease) observed when adding Cu-Phen evidences coordination. However, the results are somewhat different from those obtained at pH 10.7, where the dye is also unionized. These differences are probably due to the nature of the complexes, influenced by the copper environment at pH 6.5, which is different from that at pH 10.7, as shown by PHREEQC.

Finally, it appears that the most pronounced decrease of color was obtained at pH 10.7, which seems to meet the optimal conditions for copper-dye coordination accompanied by color-stripping effect.

At this step of the study, since UV-vis spectroscopy provided only limited information on changes of electronic structures of chromophores affected by coordination and solution environment, supplementary investigations were performed by EPR spectroscopy to study the occurrence of coordination.

#### 2.4.4. Proof of Dye-Cu-Phen Coordination

**Figure 18:**
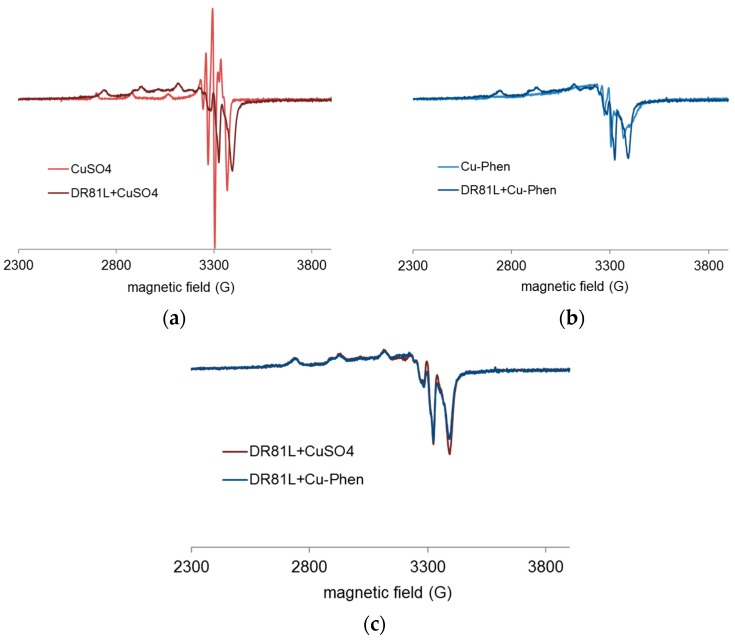
Experimental X-band EPR spectra recorded at 100 K in frozen aqueous solutions of (**a**) CuSO_4_ and CuSO_4_ + DR81 (**b**) Cu-Phen and Cu-Phen + DR81 (**c**) CuSO_4_ + DR81 and Cu-Phen + DR81, at pH 12.3, with Cu:Phen molar ratio = 1:2, DR81:Cu molar ratio = 1:1.5 and [Cu] = 1.5 mM.

**Figure 19:**
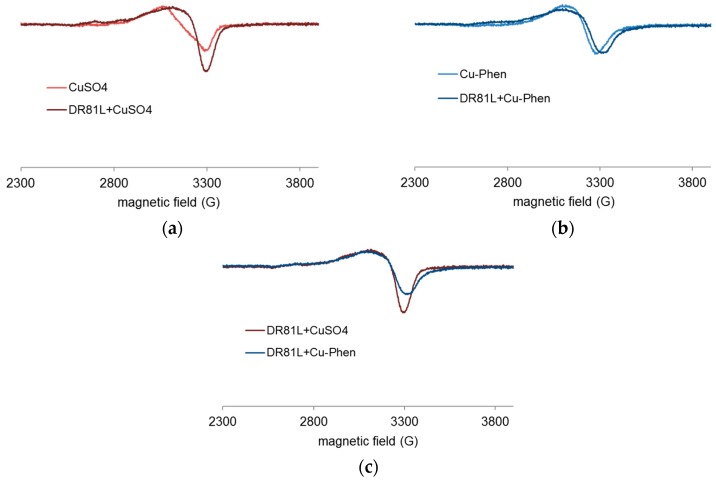
Experimental X-band EPR spectra recorded at 100 K in frozen aqueous solutions of (**a**) CuSO_4_ and CuSO_4_ + DR81 (**b**) Cu-Phen and Cu-Phen + DR81 (**c**) CuSO_4_ + DR81 and Cu-Phen + DR81, at pH 6.5, with Cu:Phen molar ratio = 1:2, DR81:Cu molar ratio = 1:1.5 and [Cu] = 1.5 mM.

EPR spectroscopy was used to study the chemical environment of copper(II), depending on the medium conditions. The analyses were conducted at pH 12.3 (strong alkaline pH), pH 10.7 (ionization of the dye), and pH 6.5 for a better comprehension of the occurring phenomena. Figure 18 and Figure 19 display EPR spectra of CuSO_4_ with and without phenanthroline (Cu:Phen molar ratio = 1:2), and in the presence of DR81 or not, recorded at pH 12.3 (Figure 18) and pH 6.5 (Figure 19).

At pH 12.3, the addition of DR81 notably modified the EPR spectrum of CuSO_4_ (Figure 18a), displaying different coupling constants and thus confirming the Cu-dye coordination. In the same manner, Figure 18b shows the Cu-dye coordination in the presence of phenanthroline. Figure 18c shows that with or without phenanthroline, copper spectra in the presence of DR81 were absolutely similar and superimposed. This observation implies that the phenanthroline ligand has been fully eliminated and replaced by the dye in the coordination sphere of the copper ion. The affinity of copper towards the dye is thus higher than that towards phenanthroline.

According to the PHREEQC simulations (without dye), under these conditions, the major species at this pH in a 1:2 complex solution would be Cu(Phen)(OH)_2_. This means that in the presence of dye, one phenanthroline would be ejected to leave space for dye coordination. In the case of copper alone, the major species without dye would be Cu(OH)_3_^−^, one hydroxyl group of which can easily be exchanged with the dye molecule. However, such a modification of the copper environment hardly affected the dye chromophore structure, as it was seen in the corresponding UV-visible spectrum of Figure 10.

Figure 19 displays the results obtained at near-neutral pH (6.5). At this pH, CuSO_4_ and Cu-Phen solutions displayed different EPR spectra. These spectra also differed from those recorded at pH 12.3. Different complexes are present depending on the pH, as predicted by PHREEQC. In the case of CuSO_4_ (Figure 19a), the addition of DR81 led to the appearance of a new EPR signature. This signature probably corresponds to a mixture of a majority of CuSO_4_ and some Cu-dye complex, as the hyperfine structure was not quite visible. A similar effect was observed in the case of Cu-Phen (Figure 19b), with a new signature probably due to a mixture of Cu-Phen and some Cu-dye complex. Although rather comparable, the signals of Figure 19c are not fully superimposed, contrarily to the equivalent signals at pH 12.3 (Figure 18c). Again, it is shown that the dye interacts strongly with copper(II). The small difference between the signals of Figure 19c is attributed to the presence of some phenanthroline in the coordination sphere of the copper ion (with Cu-Phen), which differs from the case at pH 12.3. At pH 6.5, speciation indicates that the major species with a 1:2 Cu:Phen molar ratio would be Cu(Phen)_2_^2+^, as compared to Cu(Phen)(OH)_2_ at pH 12.3. Therefore, an average of 2 phenanthroline ligands should be present around the copper atom in a Cu-Phen solution of 1.5 mM at pH 6.5. Hence, it seems unlikely that both phenanthroline ligands would be ejected to be replaced by the dye, which would easily explain the differences between the DR81 + CuSO_4_ and DR81 + Cu-Phen spectra.

When mixing DR81 to Cu-Phen, the best “color-stripping” results were obtained at pH 10.7. Therefore, it was interesting to perform a few EPR analyses at this pH, in order to check whether the dye-Cu-Phen coordination was enhanced. If so, it could be owing to speciation differences or to the non-ionized state of the dye at that pH.

**Figure 20:**
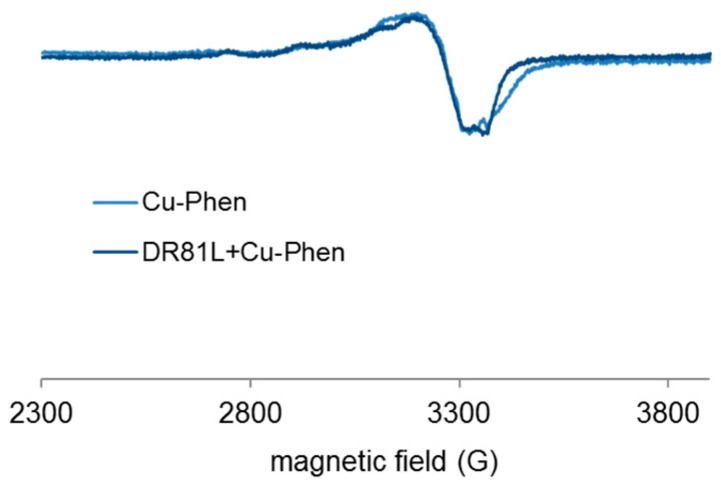
Experimental X-band EPR spectra recorded at 100 K in frozen aqueous solutions of Cu-Phen and Cu-Phen + DR81 at pH 10.7, with Cu:Phen molar ratio = 1:2, DR81:Cu molar ratio = 1:1.5 and [Cu] = 1.5 mM.

Frozen solutions of Cu-Phen and Cu-Phen + DR81 were thus analyzed at pH 10.7, as shown in Figure 20. Figure 20 shows that coordination also occurred at pH 10.7. The signals were different from those at pH 12.3 and 6.5, suggesting again a different mixture of coordinates. This is quite logical since the PHREEQC simulation gave the Cu(Phen)_3_^2+^ complex as the major species at pH 10.7 (45% of total Cu), with a relatively high amount of Cu(Phen)(OH)_2_ as well (34%).

**Figure 21:**
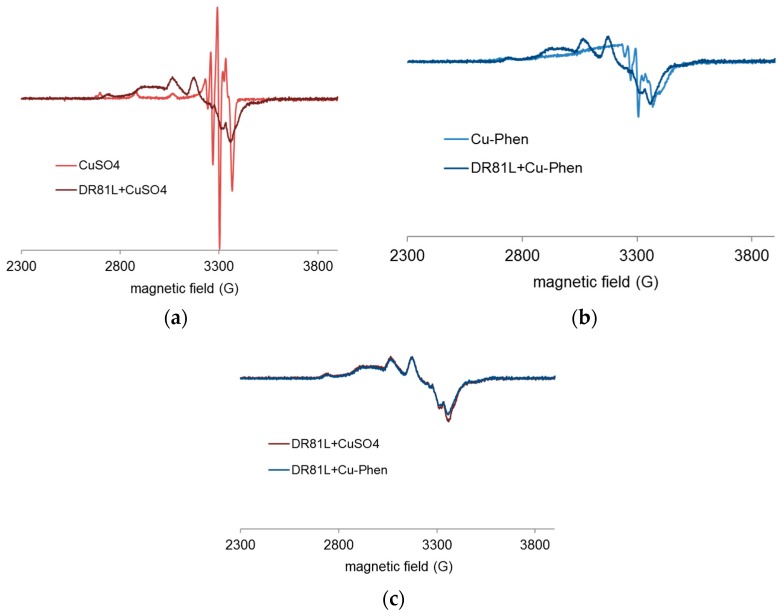
Experimental X-band EPR spectra recorded at 100 K in frozen aqueous solutions of (**a**) CuSO_4_ and CuSO_4_ + DR81 (**b**) Cu-Phen and Cu-Phen + DR81 (**c**) CuSO_4_ + DR81 and Cu-Phen + DR81, at pH 12.3, with Cu:Phen molar ratio = 1:2, DR81:Cu molar ratio = 10:1.5 and [Cu] = 1.5 mM.

In order to better understand the effect of dye addition to the copper sulfate or copper-phenanthroline solution, an excess of dye was tested: DR81:Cu molar ratio = 10:1.5 instead of 1:1.5. The results at pH 12.3 are presented in Figure 21.

**Figure 22:**
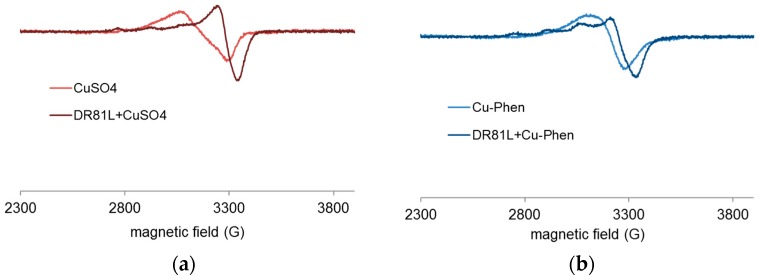

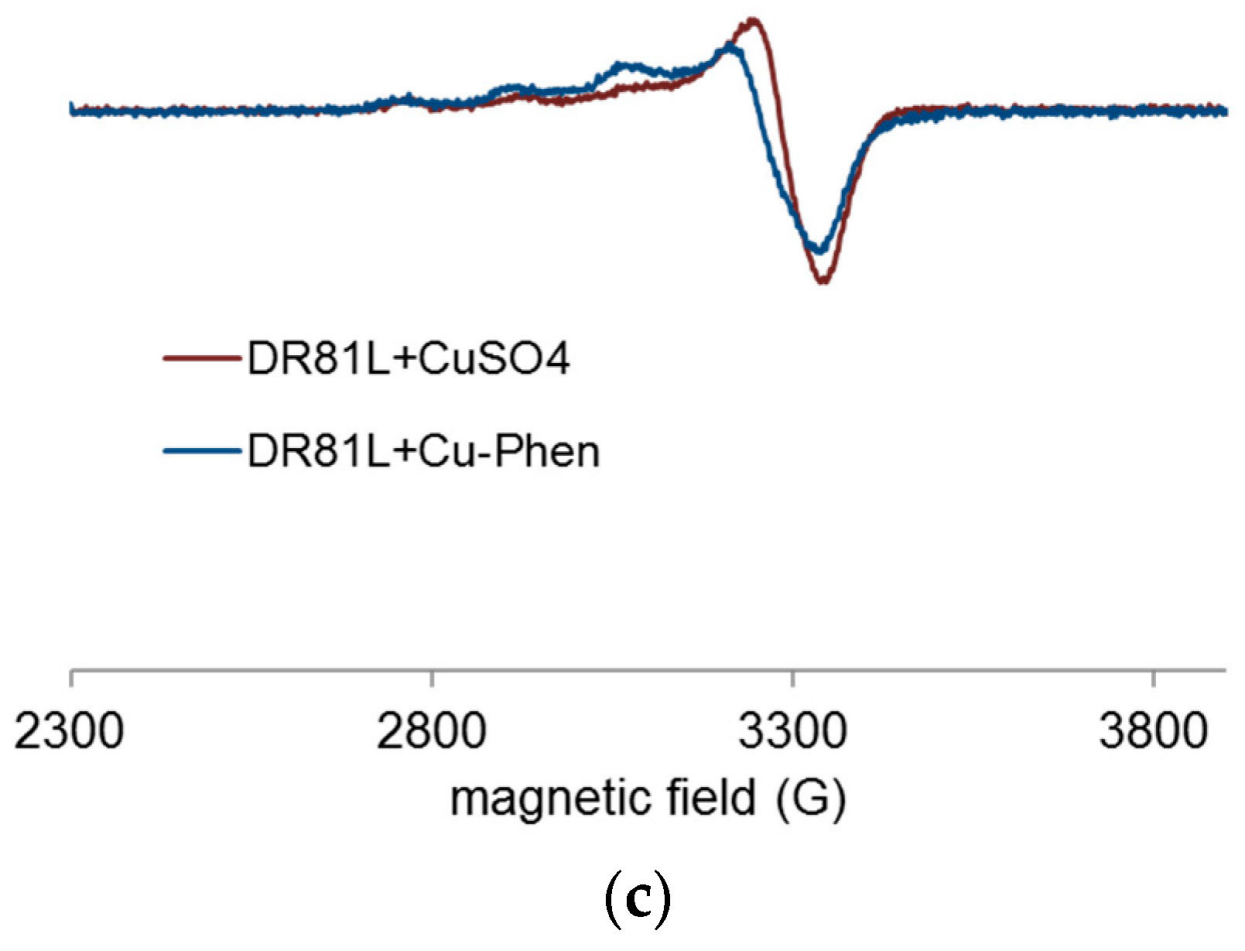
Experimental X-band EPR spectra recorded at 100 K in frozen aqueous solutions of (**a**) CuSO_4_ and CuSO_4_ + DR81 (**b**) Cu-Phen and Cu-Phen + DR81 (**c**) CuSO_4_ + DR81 and Cu-Phen + DR81, at pH 6.5, with Cu:Phen molar ratio = 1:2, DR81:Cu molar ratio = 10:1.5 and [Cu] = 1.5 mM.

Using an excess of dye, the addition of DR81 clearly structured copper’s environment, as the hyperfine structure is markedly visible in both cases (Cu in Figure 21a and Cu-Phen in Figure 21b). However, as well as with a lower amount of dye, Figure 21c shows that phenanthroline was ejected from copper’s environment in the presence of DR81: the same Cu-dye coordinate was formed in both cases. According to the signature, it was certainly a mixture of coordinates. However, this signature does not seem to exhibit the hyperfine coupling constants of CuSO_4_ or Cu-Phen. Therefore, most of the copper was probably bonded to the dye.The same experiment was repeated at pH 6.5 with an excess of DR81 (Figure 22).

At pH 6.5, again, the structuration of copper when adding DR81 was more noticeable than with a lower amount of dye. This confirms the coordinating effect between copper and DR81 also in near-neutral medium. Besides, as well as with DR81:Cu = 1:1.5, Figure 22c exhibits two different spectra. The DR81-CuSO_4_ mixture was thus different from the DR81-Cu-Phen mixture: phenanthroline was not fully replaced by the dye in copper’s coordination sphere in the second case. Finally, the coordination effect was more important with an excess of dye but the differences depending on the pH did not vary.

All these results, in which phenanthroline would not or only partly be coordinated to the copper-dye complex, corroborate the previous UV-visible spectroscopic observation, as similar “color-stripping” was observed with either CuSO_4_ or Cu-Phen, at both pH 10.7 (strong effect) and pH 12.3 (weak effect). At pH 6.5, however, Cu-Phen seemed to have a more intense hypochromic effect on the dye than CuSO_4_ alone. This is most likely due to the formation of a Phen-Cu-dye co-coordinate in the first case.

As the “color-stripping” effect was much higher at pH 10.7, it can be assumed that complexation has more or less influence on the chromophore structure of the dye. Moreover, it probably also depends on the involved coordinates and on the pH.

Actually, two cases can be distinguished:

(1) In strong alkaline conditions (ionized dye at pH 12.3) and at near neutral pH (unionized dye, pH 6.5), with or without phenanthroline, the chromophore’s electronic structure was hardly affected by copper complexation.
(2) In medium alkaline conditions, pH 10.7 (unionized dye), with or without phenanthroline, the chromophore structure was partly modified.

The EPR results also showed that the geometry around the copper ion varied as a function of pH for all three species, i.e., CuSO_4_, Cu-Phen and Cu-DR81. Besides, at near-neutral pH, some phenanthroline remained in the coordination sphere of copper, probably owing to the presence of some Phen-Cu-DR81 complex.

## 3. Materials and Methods

### 3.1. Raw Materials

The direct azo dye Carta Red 8 BL liquid (C.I. Direct Red 81, 16% *w*/*w*), DR81, was provided by Archroma (Reinach, Switzerland). Commercial chemical products of analytical or reagent grade were used: NaOH (99%, reagent grade, Carl Roth, Karlsruhe, Germany), H_2_O_2_ (35%, Carl Roth), CuSO_4_·5H_2_O (98.0%, ACS reagent, Sigma Aldrich, now Merck, Darmstadt, Germany), 1,10-phenanthroline (99.0%, Acros Organics, Geel, Belgium).

### 3.2. Stock Solution Preparation

The solvent was ultrapure water, degassed using a slight flow of nitrogen gas. The dye was diluted at a concentration of 400 µM taking into account the concentration of the dye solution given by the supplier. The Cu-Phen stock solution was composed of 1,10-phenanthroline and copper-sulfate: 1,10-phenanthroline was first ultrasonically solubilized, after what CuSO_4_·5H_2_O was added to obtain a complex concentration of 5 mM. The initial Cu:Phen molar ratio was either 1:1, 1:2 or 1:3. Considering that most of the copper was coordinated, the complex concentration was calculated as being equal to the copper concentration. The controls were prepared according to the same procedure, without phenanthroline in the case of CuSO_4_ alone and without CuSO_4_·5H_2_O in the case of phenanthroline alone.

### 3.3. Reactions

The reactions were performed with an initial dye concentration of 10 µM at room temperature (25 °C ± 2). 1 µmole of dye was mixed with the desired additives to obtain a final volume of 100 mL, in the following order: degassed ultrapure water; dye; NaOH 1 M or diluted H_2_SO_4_; CuSO_4_·5H_2_O, Phen or Cu-Phen; H_2_O_2_. A flow of nitrogen was then blown during approximately 30 s on the surface of the solution and the top of the beaker was sealed before 5 min of magnetic stirring. A sample of this solution was immediately analyzed by UV-vis spectroscopy.

The stoichiometry between Cu-Phen and the dye varied from 0.5 to 1.5 since 1 mole of complex was assumed to be able to coordinate to 1 mole of dye.

The pH of each solution was checked before reaction on a duplicate solution to avoid any pollution or air-induced oxidation of the sample to be analyzed. The final pH of the remaining solution was measured during the spectrum acquisition. It was set to 10.7 and 12.3 (alkaline and high alkaline conditions) thanks to addition of NaOH 1 M and some experiments required the addition of lower amounts of NaOH or diluted H_2_SO_4_ in order to obtain the desired pH. No significant deviation was observed between the initial and final pH.

The same protocol was applied for scientific control with the dye and copper-phenanthroline solutions alone, also analyzed by UV-vis spectroscopy.

### 3.4. Centrifugation

In case of precipitation, the samples were centrifuged with a 3-16P laboratory centrifuge (Sigma, Osterode am Harz, Germany) in order to further analyze the supernatant by UV-vis spectroscopy. The parameters were set between 3000 and 4000 rpm with a relative centrifuge force of 1730 to 3076× *g*.

### 3.5. UV-Vis Spectroscopy

The color variation of the dye solution was followed by UV-vis spectroscopy. The absorbance spectra were recorded from 200 to 800 nm on a UV-1800 UV-vis spectrophotometer (Shimadzu, Kyoto, Japan). When necessary, the samples were centrifuged beforehand. In accordance with Beer-Lambert law, the dye concentration was proportional to its absorbance at its maximum absorption wavelength. Several replicates were analyzed to verify the reproducibility of the measurements, which was useful to evaluate whether differences between two results were significant.

### 3.6. EPR Spectroscopy

Since they exhibit a d^9^ configuration, Cu(II) and its coordinates are paramagnetic species. These give a typical EPR signal depending on the coordination geometry.

X-band EPR spectra were recorded at 100 K with an EMX Plus spectrometer (Bruker, Billerica, MA, USA) equipped with an ER-4131 VT Bruker cavity for liquid nitrogen experiments. Indeed, these EPR analyses were conducted on frozen samples. This allows to obtain a random collection of paramagnets and to improve the spectrum resolution.

The instrument settings were the following: receiver gain 30 dB, modulation amplitude 4 G, attenuation 10 dB. The tested samples were aqueous solutions of CuSO_4_ and Cu-Phen, with or without DR81, and with or without sodium hydroxide, leading to pH 12.3–12.5 or pH 6.5–7, respectively. The concentrations were the following: 1 mM DR81, 1.5 mM CuSO_4_·5H_2_O, and Cu:Phen molar ratio = 1:2.

## 4. Conclusions

In conclusion, the results showed that whatever the pH, the addition of Cu-Phen complexes can reduce the color of a Direct Red 81 aqueous solution. This apparent color-stripping effect would be due to the coordination of copper with the dye after total or partial release of the phenanthroline ligand, which was evidenced by EPR spectroscopy. Interestingly, at pH 6.5, some phenanthroline remained in the coordination sphere of copper, whereas at alkaline pH, it seemed to be ejected.

The complexation of copper with Direct Red 81 is certainly responsible for the decrease of its absorbance in the visible range, up to 50% color-stripping depending on the pH, under the concentration conditions used, although this absorbance could not be totally cancelled. This phenomenon seemed to be strongly correlated with the nature of the Cu-Phen species which in turn are dependent on the pH. The best color-stripping results were obtained at medium alkalinity (pH 10.7), with the Cu(Phen)(OH)_2_ species, leading to a hydroxylated dye-copper complex after phenanthroline ejection.

The dye-complexing effect of Cu-Phen species may be interesting for dye effluent treatment, especially in case of already alkaline effluents. It might be used as an alternative to physicochemical techniques, such as chemical oxidation, chemical reduction, adsorption or light-illumination treatments, to decolorize colored wastewaters.

## Figures and Tables

**Table 1:** Predicted abundances (expressed as percentages of total Cu) of the different species at pH 6.5, 10.7 and 12.3, for Cu:Phen molar ratios = 1:0, 1:1, 1:2 and 1:3, with an initial CuSO_4_ concentration of 15 µM at 25 °C, assuming no precipitation occurrence. The results are given as molar percentages of total [Cu].

Cu:Phen	1:0			1:1			1:2			1:3		
pH	6.5	10.7	12.3	6.5	10.7	12.3	6.5	10.7	12.3	6.5	10.7	12.3
Cu^2+^	90.2	ε	ε	5.1	ε	ε	ε	ε	ε	ε	ε	ε
CuOH^+^	8.8	0.5	ε	0.5	ε	ε	ε	ε	ε	ε	ε	ε
Cu(OH)_2_	0.1	48.1	1.6	ε	1.7	0.3	ε	0.1	0.1	ε	ε	ε
CuSO_4_	0.3	ε	ε	ε	ε	ε	ε	ε	ε	ε	ε	ε
Cu_2_(OH)_2_^2+^	0.6	ε	ε	ε	ε	ε	ε	ε	ε	ε	ε	ε
Cu(OH)_3_^−^	ε ^a^	51.2	77.4	ε	1.8	14.5	ε	0.1	3.1	ε	ε	1.6
Cu(OH)_4_^2−^	ε	0.2	21.0	ε	ε	3.9	ε	ε	0.8	ε	ε	0.4
Cu(Phen)^2+^	/	/	/	84.3	ε	ε	15.9	ε	ε	1.3	ε	ε
Cu(Phen)_2_^2+^	/	/	/	5.5	ε	ε	72.8	0.6	ε	45.8	1.1	ε
Cu(Phen)_3_^2+^	/	/	/	ε	ε	ε	10.6	1.4	ε	52.8	4.9	ε
(CuPhenOH)^+^	/	/	/	4.1	5.8	0.2	0.8	5.9	0.2	0.1	5.7	0.2
(CuPhenOH)_2_^2+^	/	/	/	0.4	0.9	ε	ε	0.9	ε	ε	0.8	ε
CuPhen(OH)_2_	/	/	/	ε	89.7	81.2	ε	91.1	95.9	ε	87.4	97.7
Phen	/	/	/	0.1	3.5	18.7	6.1	96.8	104.0	48	189	202

^a^ ε represents a negligible percentage (below 0.1%).

**Table 2:** Predicted abundances (expressed as percentages of total Cu) of the different species at pH 6.5, 10.7 and 12.3, for Cu:Phen molar ratios = 1:0, 1:1, 1:2 and 1:3, with an initial CuSO_4_ concentration of 15 µM at 25 °C, assuming the occurrence of solids precipitation. The results are given as molar percentages of total [Cu].

Cu:Phen	1:0			1:1			1:2			1:3		
pH	6.5	10.7	12.3	6.5	10.7	12.3	6.5	10.7	12.3	6.5	10.7	12.3
Cu^2+^	29.9	ε	ε	5.1	ε	ε	ε	ε	ε	ε	ε	ε
CuOH^+^	3.0	ε	ε	0.5	ε	ε	ε	ε	ε	ε	ε	ε
Cu(OH)_2_	ε ^a^	ε	ε	ε	ε	ε	ε	ε	ε	ε	ε	ε
CuSO_4_	0.1	ε	ε	ε	ε	ε	ε	ε	ε	ε	ε	ε
Cu_2_(OH)_2_^2+^	0.1	ε	ε	ε	ε	ε	ε	ε	ε	ε	ε	ε
Cu(OH)_3_^−^	ε	ε	0.9	ε	ε	0.9	ε	ε	0.9	ε	ε	0.9
Cu(OH)_4_^2−^	ε	ε	0.3	ε	ε	0.3	ε	ε	0.3	ε	ε	0.3
Cu(Phen)^2+^	/	/	/	84.3	ε	ε	15.9	ε	ε	1.3	ε	ε
Cu(Phen)_2_^2+^	/	/	/	5.5	0.1	ε	72.8	0.4	ε	45.8	0.9	ε
Cu(Phen)_3_^2+^	/	/	/	ε	0.2	ε	10.6	1.5	ε	52.8	4.6	ε
(CuPhenOH)^+^	/	/	/	4.1	1.4	ε	0.8	2.7	0.1	0.1	4.0	0.1
(CuPhenOH)_2_^2+^	/	/	/	0.4	ε	ε	ε	0.2	ε	ε	0.4	ε
CuPhen(OH)_2_	/	/	/	ε	21.3	21.7	ε	41.7	43.5	ε	60.9	65.2
Phen	/	/	/	ε	76.5	78.2	6.1	150.1	156.5	48.5	219.1	234.7
Tenorite	66.9	100.0	98.8	ε	77.0	77.0	ε	53.4	55.3	ε	29.2	33.5
Solids-Cu	66.9	100.0	98.8	ε	77.0	77.0	ε	53.4	55.3	ε	29.2	33.5

^a^ ε represents a negligible percentage (below 0.1%).
